# Bis[2-(8-quinolylimino­meth­yl)phenolato-κ^3^ *N*,*N*′,*O*]iron(III) azide

**DOI:** 10.1107/S1600536810002783

**Published:** 2010-01-27

**Authors:** Yoshihiro Kojima, Kazuya Kato, Yuuki Yamamoto, Katsuya Inoue, Shinya Hayami

**Affiliations:** a Department of Chemistry, Graduate School of Science, Hiroshima University, 1-3-1 Kagamiyama, Higashi-Hiroshima 739-8526, Japan; b Department of Chemistry, Faculty of Science, Kumamoto University, 2-39-1 Kurokami, Kumamoto 860-8555, Japan; c Department of Chemistry and Institute for Advanced Materials Research, Hiroshima University, 1-3-1 Kagamiyama, Higashi-Hiroshima 739-8526, Japan; d Department of Chemistry, Graduate School of Science and Technology, Kumamoto University, 2-39-1 Kurokami, Kumamoto 860-8555, Japan

## Abstract

The title compound, [Fe(C_16_H_11_N_2_O)_2_]N_3_, consists of a [Fe(qsal)_2_]^+^ cation [Hqsal = *N*-(8-quinol­yl)salicylaldimine] and an azide anion. The Fe^III^ ion, lying on a twofold rotation axis, is coordinated by four N atoms and two O atoms from two tridentate qsal ligands in an octa­hedral geometry. The mol­ecules are connected into a three-dimensional network by inter­molecular C—H⋯N and C—H⋯O inter­actions. π–π inter­actions [inter­planar distance = 3.58 (1) Å] between the quinoline rings of adjacent mol­ecules further stabilize the crystal structure.

## Related literature

For Fe(III) complexes with qsal ligands, see: Hayami *et al.* (2001 ▶); Takahashi *et al.* (2006 ▶). For bond lengths in Fe(III) complexes, see: Nihei *et al.* (2007 ▶).
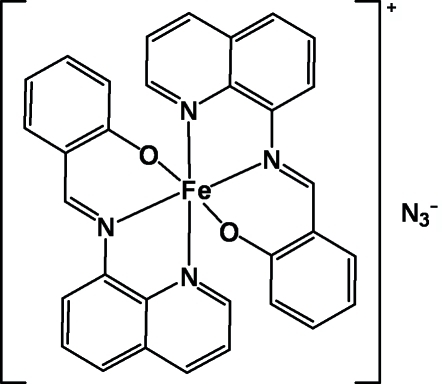


## Experimental

### 

#### Crystal data

[Fe(C_16_H_11_N_2_O)_2_]N_3_
*M* *_r_* = 592.42
Monoclinic, 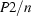

*a* = 11.3717 (8) Å
*b* = 10.1190 (8) Å
*c* = 11.7734 (6) Å
β = 109.3542 (15)°
*V* = 1278.21 (15) Å^3^
*Z* = 2
Mo *K*α radiation
μ = 0.64 mm^−1^
*T* = 200 K
0.50 × 0.20 × 0.20 mm

#### Data collection

Rigaku R-AXIS RAPID diffractometer
Absorption correction: multi-scan (*ABSCOR*; Higashi, 1995 ▶) *T*_min_ = 0.741, *T*_max_ = 0.883
11530 measured reflections
2929 independent reflections
2556 reflections with *I* > 2σ(*I*)
*R*_int_ = 0.026

#### Refinement

*R*[*F* ^2^ > 2σ(*F* ^2^)] = 0.031
*wR*(*F* ^2^) = 0.104
*S* = 0.86
2929 reflections
191 parameters
H-atom parameters constrained
Δρ_max_ = 0.34 e Å^−3^
Δρ_min_ = −0.39 e Å^−3^

### 

Data collection: *PROCESS-AUTO* (Rigaku, 1998 ▶); cell refinement: *PROCESS-AUTO*; data reduction: *CrystalClear* (Rigaku, 2002 ▶); program(s) used to solve structure: *SHELXS97* (Sheldrick, 2008 ▶); program(s) used to refine structure: *SHELXL97* (Sheldrick, 2008 ▶); molecular graphics: *Yadokari-XG* (Wakita, 2000 ▶); software used to prepare material for publication: *SHELXL97*.

## Supplementary Material

Crystal structure: contains datablocks I, global. DOI: 10.1107/S1600536810002783/hy2271sup1.cif

Structure factors: contains datablocks I. DOI: 10.1107/S1600536810002783/hy2271Isup2.hkl

Additional supplementary materials:  crystallographic information; 3D view; checkCIF report

## Figures and Tables

**Table 1:** Selected bond lengths (Å)

Fe1—O1	1.8648 (11)
Fe1—N1	1.9347 (13)
Fe1—N2	1.9680 (12)

**Table 2:** Hydrogen-bond geometry (Å, °)

*D*—H⋯*A*	*D*—H	H⋯*A*	*D*⋯*A*	*D*—H⋯*A*
C9—H9⋯O1^i^	0.95	2.70	3.565 (2)	151
C15—H15⋯N4^ii^	0.95	2.45	3.299 (2)	149

Symmetry codes: (i) 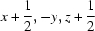; (ii) 
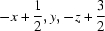
.

**Fig. 1.**
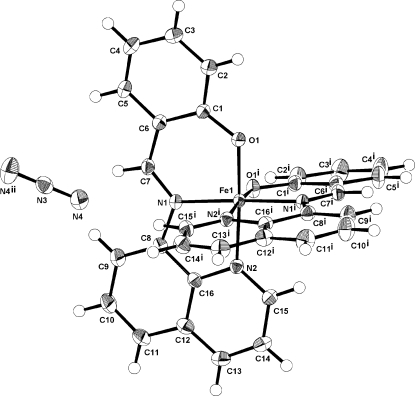
The molecular structure of the title compound drawn with the 50% probability displacement ellipsoids. [Symmetry codes: (i) 1/2-x, y, 3/2-z; (ii) 1/2-x, y, 5/2-z.]

## supplementary crystallographic information

### Comment

Fe(III) complexes with the qsal ligands [Hqsal = *N*-(8-quinolyl)salicylaldimine] were reported previously (Hayami *et al*., 2001; Takahashi *et al.*, 2006). It was showed that the spin states of the complexes can be tuned by the different counter anions. Some of the complexes were observed spin crossover or spin transition. The title compound (Fig. 1) does not show both spin-crossover behaviors but is in the low-spin state at room temperature. In the complex, Fe1—O1, Fe1—N1 and Fe1—N2 bond lengths (Table 1) are close to those for other low-spin Fe(III) complexes (Nihei *et al*., 2007).

In addition, many intermolecular interactions are observed in the crystal structure. The intermolecular C—H···N hydrogen bond (involving quinoline ring H15 and the azide N4), C—H···O hydrogen bond (involving quinoline ring H9 and phenolate O1) (Table 2), and π–π interaction [interplanar distance = 3.58 (1) Å] between the quinoline rings of adjacent molecules link the molecules and provide stability into the crystal structure.

### Experimental

Hqsal and [Fe(qsal)_2_]Cl were prepared from 8-aminoquinoline and salicylaldehyde according to the method described previously (Hayami *et al.*, 2001). The title compound was prepared by slow addition of a MeOH solution (30 ml) containing [Fe(qsal)_2_]Cl (0.5 mmol) to a MeOH solution (30 ml) containing an excess of NaN_3_ (2 mmol).

### Refinement

All H atoms were positioned geometrically (C—H = 0.95 Å) and refined as riding atoms, with *U*_iso_(H) = 1.2*U*_eq_(C).

### Crystal data

**Table:**

[Fe(C_16_H_11_N_2_O)_2_]N_3_	*F*(000) = 610
*M**_r_* = 592.42	*D*_x_ = 1.539 Mg m^−^^3^
Monoclinic, *P*2/*n*	Mo *K*α radiation, λ = 0.71069 Å
Hall symbol: -P 2yac	Cell parameters from 11530 reflections
*a* = 11.3717 (8) Å	θ = 1.8–27.5°
*b* = 10.1190 (8) Å	µ = 0.64 mm^−^^1^
*c* = 11.7734 (6) Å	*T* = 200 K
β = 109.3542 (15)°	Block, black
*V* = 1278.21 (15) Å^3^	0.50 × 0.20 × 0.20 mm
*Z* = 2	

### Data collection

**Table:**

Rigaku R-AXIS RAPID diffractometer	2929 independent reflections
Radiation source: rotation anode	2556 reflections with *I* > 2σ(*I*)
graphite	*R*_int_ = 0.026
ω scans	θ_max_ = 27.5°, θ_min_ = 2.0°
Absorption correction: multi-scan (*ABSCOR*; Higashi, 1995)	*h* = 0→14
*T*_min_ = 0.741, *T*_max_ = 0.883	*k* = 0→13
11530 measured reflections	*l* = −15→14

### Refinement

**Table:**

Refinement on *F*^2^	Primary atom site location: structure-invariant direct methods
Least-squares matrix: full	Secondary atom site location: difference Fourier map
*R*[*F*^2^ > 2σ(*F*^2^)] = 0.031	Hydrogen site location: inferred from neighbouring sites
*wR*(*F*^2^) = 0.104	H-atom parameters constrained
*S* = 0.86	*w* = 1/[σ^2^(*F*_o_^2^) + (0.1*P*)^2^] where *P* = (*F*_o_^2^ + 2*F*_c_^2^)/3
2929 reflections	(Δ/σ)_max_ < 0.001
191 parameters	Δρ_max_ = 0.34 e Å^−^^3^
0 restraints	Δρ_min_ = −0.39 e Å^−^^3^

### Fractional atomic coordinates and isotropic or equivalent isotropic displacement parameters (Å^2^)

**Table:**

	*x*	*y*	*z*	*U*_iso_*/*U*_eq_	
Fe1	0.2500	0.12760 (3)	0.7500	0.01593 (12)	
O1	0.13778 (10)	0.00441 (11)	0.77255 (10)	0.0230 (2)	
N1	0.35553 (12)	0.13158 (12)	0.91699 (11)	0.0181 (3)	
N2	0.36114 (11)	0.27008 (12)	0.73375 (11)	0.0184 (3)	
N3	0.2500	0.28128 (19)	1.2500	0.0279 (4)	
N4	0.26580 (17)	0.28132 (18)	1.15630 (15)	0.0433 (4)	
C1	0.14342 (14)	−0.05156 (14)	0.87550 (13)	0.0211 (3)	
C2	0.04475 (17)	−0.13609 (16)	0.87415 (16)	0.0286 (4)	
H2	−0.0224	−0.1491	0.8012	0.034*	
C3	0.04411 (18)	−0.20050 (19)	0.97754 (16)	0.0352 (4)	
H3	−0.0236	−0.2569	0.9745	0.042*	
C4	0.1411 (2)	−0.1841 (2)	1.08613 (16)	0.0395 (5)	
H4	0.1409	−0.2307	1.1561	0.047*	
C5	0.23689 (18)	−0.09964 (19)	1.09040 (15)	0.0321 (4)	
H5	0.3022	−0.0865	1.1646	0.038*	
C6	0.24043 (15)	−0.03176 (15)	0.98671 (14)	0.0230 (3)	
C7	0.34174 (14)	0.05762 (15)	1.00231 (13)	0.0218 (3)	
H7	0.4031	0.0636	1.0799	0.026*	
C8	0.45447 (14)	0.22483 (14)	0.94235 (13)	0.0207 (3)	
C9	0.54613 (16)	0.24933 (17)	1.05052 (15)	0.0293 (4)	
H9	0.5493	0.1992	1.1197	0.035*	
C10	0.63516 (17)	0.3487 (2)	1.05877 (17)	0.0355 (4)	
H10	0.6988	0.3639	1.1337	0.043*	
C11	0.63210 (16)	0.42458 (18)	0.96008 (16)	0.0323 (4)	
H11	0.6924	0.4920	0.9679	0.039*	
C12	0.53937 (15)	0.40166 (16)	0.84810 (15)	0.0252 (3)	
C13	0.52736 (15)	0.47520 (17)	0.74226 (16)	0.0291 (4)	
H13	0.5829	0.5460	0.7443	0.035*	
C14	0.43587 (15)	0.44350 (16)	0.63807 (15)	0.0272 (3)	
H14	0.4270	0.4925	0.5669	0.033*	
C15	0.35431 (14)	0.33827 (16)	0.63543 (14)	0.0221 (3)	
H15	0.2926	0.3153	0.5613	0.027*	
C16	0.45197 (13)	0.30040 (15)	0.83972 (13)	0.0194 (3)	

### Atomic displacement parameters (Å^2^)

**Table:**

	*U*^11^	*U*^22^	*U*^33^	*U*^12^	*U*^13^	*U*^23^
Fe1	0.01757 (17)	0.01657 (17)	0.01266 (17)	0.000	0.00365 (11)	0.000
O1	0.0252 (5)	0.0240 (5)	0.0169 (5)	−0.0063 (4)	0.0032 (4)	0.0021 (4)
N1	0.0187 (6)	0.0199 (6)	0.0149 (6)	0.0007 (4)	0.0045 (5)	−0.0004 (4)
N2	0.0174 (5)	0.0205 (6)	0.0174 (6)	0.0000 (5)	0.0059 (5)	0.0001 (4)
N3	0.0242 (9)	0.0256 (10)	0.0281 (10)	0.000	0.0009 (8)	0.000
N4	0.0472 (10)	0.0516 (10)	0.0297 (9)	−0.0174 (8)	0.0110 (7)	−0.0039 (7)
C1	0.0265 (7)	0.0198 (7)	0.0175 (7)	−0.0006 (6)	0.0079 (6)	0.0011 (5)
C2	0.0292 (8)	0.0309 (9)	0.0244 (8)	−0.0087 (7)	0.0071 (7)	0.0009 (6)
C3	0.0408 (10)	0.0377 (9)	0.0293 (9)	−0.0159 (8)	0.0147 (8)	0.0011 (7)
C4	0.0536 (11)	0.0434 (11)	0.0224 (8)	−0.0161 (9)	0.0138 (8)	0.0049 (7)
C5	0.0408 (10)	0.0356 (9)	0.0166 (8)	−0.0079 (8)	0.0052 (7)	0.0038 (6)
C6	0.0286 (8)	0.0216 (7)	0.0188 (7)	−0.0012 (6)	0.0080 (6)	0.0010 (6)
C7	0.0247 (7)	0.0223 (7)	0.0155 (7)	0.0008 (6)	0.0027 (6)	0.0006 (5)
C8	0.0195 (6)	0.0215 (7)	0.0199 (7)	−0.0003 (5)	0.0049 (5)	−0.0024 (5)
C9	0.0290 (8)	0.0341 (9)	0.0215 (8)	−0.0055 (7)	0.0039 (6)	−0.0012 (6)
C10	0.0284 (9)	0.0451 (10)	0.0254 (9)	−0.0118 (8)	−0.0012 (7)	−0.0045 (7)
C11	0.0282 (8)	0.0346 (9)	0.0315 (9)	−0.0123 (7)	0.0064 (7)	−0.0049 (7)
C12	0.0217 (7)	0.0249 (8)	0.0288 (9)	−0.0027 (6)	0.0080 (6)	−0.0026 (6)
C13	0.0280 (8)	0.0266 (8)	0.0346 (9)	−0.0066 (7)	0.0129 (7)	0.0012 (7)
C14	0.0283 (8)	0.0263 (8)	0.0295 (8)	−0.0013 (6)	0.0128 (7)	0.0066 (6)
C15	0.0204 (7)	0.0248 (7)	0.0213 (7)	0.0005 (6)	0.0071 (6)	0.0035 (6)
C16	0.0172 (6)	0.0203 (7)	0.0202 (7)	−0.0001 (5)	0.0054 (5)	−0.0020 (5)

### Geometric parameters (Å, °)

**Table:**

Fe1—O1	1.8648 (11)	C5—H5	0.9500
Fe1—N1	1.9347 (13)	C6—C7	1.428 (2)
Fe1—N2	1.9680 (12)	C7—H7	0.9500
O1—C1	1.3201 (17)	C8—C9	1.375 (2)
N1—C7	1.3031 (19)	C8—C16	1.422 (2)
N1—C8	1.4222 (19)	C9—C10	1.407 (2)
N2—C15	1.3276 (19)	C9—H9	0.9500
N2—C16	1.3650 (18)	C10—C11	1.384 (3)
N3—N4	1.1750 (17)	C10—H10	0.9500
N3—N4^i^	1.1750 (17)	C11—C12	1.408 (2)
C1—C2	1.407 (2)	C11—H11	0.9500
C1—C6	1.419 (2)	C12—C13	1.418 (2)
C2—C3	1.383 (2)	C12—C16	1.408 (2)
C2—H2	0.9500	C13—H13	0.9500
C3—H3	0.9500	C14—C13	1.358 (2)
C4—C3	1.394 (3)	C15—C14	1.406 (2)
C4—C5	1.372 (3)	C14—H14	0.9500
C4—H4	0.9500	C15—H15	0.9500
C5—C6	1.413 (2)		
			
O1—Fe1—O1^ii^	96.10 (7)	C4—C5—H5	119.3
O1—Fe1—N1	95.31 (5)	C6—C5—H5	119.3
O1—Fe1—N1^ii^	86.29 (5)	C1—C6—C5	119.50 (14)
O1^ii^—Fe1—N1	86.29 (5)	C1—C6—C7	123.74 (14)
O1^ii^—Fe1—N1^ii^	95.31 (5)	C5—C6—C7	116.72 (14)
O1—Fe1—N2	174.51 (5)	N1—C7—C6	124.24 (14)
O1—Fe1—N2^ii^	89.09 (5)	N1—C7—H7	117.9
O1^ii^—Fe1—N2	89.09 (5)	C6—C7—H7	117.9
O1^ii^—Fe1—N2^ii^	174.51 (5)	N1—C8—C9	128.20 (14)
N1^ii^—Fe1—N1	177.62 (7)	N1—C8—C16	112.74 (13)
N1—Fe1—N2	83.19 (5)	C9—C8—C16	119.05 (14)
N1—Fe1—N2^ii^	95.05 (5)	C8—C9—C10	120.01 (16)
N1^ii^—Fe1—N2	95.05 (5)	C8—C9—H9	120.0
N1^ii^—Fe1—N2^ii^	83.19 (5)	C10—C9—H9	120.0
N2^ii^—Fe1—N2	85.79 (7)	C9—C10—C11	121.43 (16)
Fe1—O1—C1	126.18 (10)	C9—C10—H10	119.3
Fe1—N1—C7	125.36 (11)	C11—C10—H10	119.3
Fe1—N1—C8	114.10 (10)	C10—C11—C12	119.83 (16)
C7—N1—C8	120.54 (13)	C10—C11—H11	120.1
Fe1—N2—C15	127.71 (10)	C12—C11—H11	120.1
Fe1—N2—C16	113.05 (9)	C11—C12—C13	124.08 (16)
C15—N2—C16	119.21 (13)	C11—C12—C16	118.54 (15)
N4—N3—N4^i^	180.0 (3)	C13—C12—C16	117.39 (15)
O1—C1—C2	117.04 (14)	C12—C13—C14	119.44 (15)
O1—C1—C6	124.95 (14)	C14—C13—H13	120.3
C2—C1—C6	118.00 (14)	C12—C13—H13	120.3
C1—C2—C3	120.89 (16)	C13—C14—C15	120.09 (15)
C1—C2—H2	119.6	C13—C14—H14	120.0
C3—C2—H2	119.6	C15—C14—H14	120.0
C2—C3—C4	121.17 (16)	N2—C15—C14	121.80 (14)
C2—C3—H3	119.4	N2—C15—H15	119.1
C4—C3—H3	119.4	C14—C15—H15	119.1
C3—C4—C5	119.02 (16)	N2—C16—C12	122.03 (14)
C5—C4—H4	120.5	N2—C16—C8	116.85 (13)
C3—C4—H4	120.5	C8—C16—C12	121.12 (14)
C4—C5—C6	121.37 (16)		
			
O1^ii^—Fe1—O1—C1	86.71 (12)	C15—N2—C16—C12	−0.3 (2)
N1—Fe1—O1—C1	−0.11 (13)	O1—C1—C2—C3	178.95 (16)
N1^ii^—Fe1—O1—C1	−178.34 (13)	C6—C1—C2—C3	−1.6 (3)
O1—Fe1—N1—C7	3.95 (13)	O1—C1—C6—C5	−178.78 (16)
O1^ii^—Fe1—N1—C7	−91.84 (13)	O1—C1—C6—C7	3.5 (2)
O1—Fe1—N1—C8	−176.17 (10)	C2—C1—C6—C5	1.9 (2)
O1^ii^—Fe1—N1—C8	88.04 (10)	C2—C1—C6—C7	−175.91 (15)
N2—Fe1—N1—C7	178.64 (13)	C1—C2—C3—C4	−0.2 (3)
N2^ii^—Fe1—N1—C7	93.51 (13)	C2—C3—C4—C5	1.7 (3)
N2—Fe1—N1—C8	−1.49 (10)	C3—C4—C5—C6	−1.5 (3)
N2^ii^—Fe1—N1—C8	−86.62 (10)	C4—C5—C6—C1	−0.3 (3)
O1^ii^—Fe1—N2—C15	95.66 (13)	C4—C5—C6—C7	177.60 (17)
N1—Fe1—N2—C15	−177.97 (13)	C1—C6—C7—N1	0.8 (2)
N1^ii^—Fe1—N2—C15	0.41 (13)	C5—C6—C7—N1	−177.02 (15)
N2^ii^—Fe1—N2—C15	−82.36 (13)	N1—C8—C9—C10	−178.44 (16)
O1^ii^—Fe1—N2—C16	−86.28 (10)	C16—C8—C9—C10	0.5 (2)
N1—Fe1—N2—C16	0.09 (10)	N1—C8—C16—N2	−2.56 (19)
N1^ii^—Fe1—N2—C16	178.47 (10)	N1—C8—C16—C12	177.32 (13)
N2^ii^—Fe1—N2—C16	95.70 (11)	C9—C8—C16—N2	178.36 (14)
Fe1—O1—C1—C2	176.17 (11)	C9—C8—C16—C12	−1.8 (2)
Fe1—O1—C1—C6	−3.2 (2)	C8—C9—C10—C11	0.8 (3)
Fe1—N1—C7—C6	−4.7 (2)	C9—C10—C11—C12	−0.9 (3)
C8—N1—C7—C6	175.48 (14)	C10—C11—C12—C13	179.10 (17)
Fe1—N1—C8—C9	−178.46 (14)	C10—C11—C12—C16	−0.4 (3)
Fe1—N1—C8—C16	2.56 (16)	C11—C12—C13—C14	178.80 (17)
C7—N1—C8—C9	1.4 (2)	C16—C12—C13—C14	−1.7 (2)
C7—N1—C8—C16	−177.56 (13)	C11—C12—C16—N2	−178.43 (15)
Fe1—N2—C15—C14	176.08 (11)	C11—C12—C16—C8	1.7 (2)
C16—N2—C15—C14	−1.9 (2)	C13—C12—C16—N2	2.1 (2)
Fe1—N2—C16—C8	1.35 (16)	C13—C12—C16—C8	−177.80 (14)
Fe1—N2—C16—C12	−178.52 (11)	C12—C13—C14—C15	−0.3 (3)
C15—N2—C16—C8	179.59 (13)	C13—C14—C15—N2	2.2 (2)

Symmetry codes: (i) −*x*+1/2, *y*, −*z*+5/2; (ii) −*x*+1/2, *y*, −*z*+3/2.

### Hydrogen-bond geometry (Å, °)

**Table:**

*D*—H···*A*	*D*—H	H···*A*	*D*···*A*	*D*—H···*A*
C9—H9···O1^iii^	0.95	2.70	3.565 (2)	151
C15—H15···N4^ii^	0.95	2.45	3.299 (2)	149

Symmetry codes: (iii) *x*+1/2, −*y*, *z*+1/2; (ii) −*x*+1/2, *y*, −*z*+3/2.
